# Routing Protocols in Wireless Sensor Networks

**DOI:** 10.3390/s91108399

**Published:** 2009-10-26

**Authors:** Luis Javier García Villalba, Ana Lucila Sandoval Orozco, Alicia Triviño Cabrera, Cláudia Jacy Barenco Abbas

**Affiliations:** Group of Analysis, Security and Systems (GASS), Department of Software Engineering and Artificial Intelligence, School of Computer Science, Office 431, Complutense University of Madrid (UCM), Calle Profesor José García Santesmases s/n, Ciudad Universitaria, 28040 Madrid, Spain; E-Mails: asandoval@fdi.ucm.es (A.S.); atrica@fdi.ucm.es (A.T.); cbarenco@fdi.ucm.es (C.B.)

**Keywords:** routing protocol, wireless sensor network

## Abstract

The applications of wireless sensor networks comprise a wide variety of scenarios. In most of them, the network is composed of a significant number of nodes deployed in an extensive area in which not all nodes are directly connected. Then, the data exchange is supported by multihop communications. Routing protocols are in charge of discovering and maintaining the routes in the network. However, the appropriateness of a particular routing protocol mainly depends on the capabilities of the nodes and on the application requirements. This paper presents a review of the main routing protocols proposed for wireless sensor networks. Additionally, the paper includes the efforts carried out by Spanish universities on developing optimization techniques in the area of routing protocols for wireless sensor networks.

## 1. Introduction

Wireless Sensor Networks (WSN) are intended for monitoring an environment. The main task of a wireless sensor node is to sense and collect data from a certain domain, process them and transmit it to the sink where the application lies. However, ensuring the direct communication between a sensor and the sink may force nodes to emit their messages with such a high power that their resources could be quickly depleted. Therefore, the collaboration of nodes to ensure that distant nodes communicate with the sink is a requirement. In this way, messages are propagated by intermediate nodes so that a route with multiple links or hops to the sink is established.

Taking into account the reduced capabilities of sensors, the communication with the sink could be initially conceived without a routing protocol. With this premise, the flooding algorithm stands out as the simplest solution. In this algorithm, the transmitter broadcasts the data which are consecutively retransmitted in order to make them arrive at the intended destination. However, its simplicity brings about significant drawbacks. Firstly, an implosion is detected because nodes redundantly receive multiple copies of the same data message. Then, as the event may be detected by several nodes in the affected area, multiple data messages containing similar information are introduced into the network. Moreover, the nodes do not take into account their resources to limit their functionalities.

One optimization relies on the gossiping algorithm [1]. Gossiping avoids implosion as the sensor transmits the message to a selected neighbor instead of informing all its neighbors as in the classical flooding algorithm. However, overlap and resource blindness are still present. Furthermore, these inconveniences are highlighted when the number of nodes in the network increases.

Due to the deficiencies of the previous strategies, routing protocols become necessary in wireless sensor networks. Nevertheless, the inclusion of a routing protocol in a wireless sensor network is not a trivial task. One of the main limitations is the identification of nodes. Since wireless sensor networks are formed by a significant number of nodes, the manual assignation of unique identifiers becomes infeasible [2]. The use of potentially unique identifier such as the MAC (Medium Access Control) address or the GPS coordinates is not recommended as it forces a significant payload in the messages [3]. However, this drawback is easily overcome in wireless sensor networks since an IP address is not required to identify the destination node of a specific packet. In fact, attribute-based addressing fits better with the specificities of wireless sensor networks. In this case, an attribute such as node location and sensor type is used to identify the final destination.

Once nodes are identified, routing protocols are in charge of constructing and maintaining routes between distant nodes. The different ways in which routing protocols operate make them appropriate for certain applications.

In the related literature, there are plenty of proposals concerning routing algorithms in wireless sensor networks. This paper aims at describing the most relevant ones in order to facilitate the understanding of the different routing techniques that could be applied into wireless sensor networks. Specifically, the paper explains some attributed-based, geographic, hierarchical and multipath routing protocols. The most significant Spanish proposals are also described.

The rest of the paper is structured as follows. Section 2 shows the basic communication paradigms that wireless sensor networks follow while Section 3 describes the main design constraints that routing protocols must face in wireless sensor networks. In Section 4, we present the most popular classification schemes for routing protocols in this kind of networks. Section 5 outlines the optimization procedures adopted by these routing protocols. The application of these techniques leads to attribute-based, geographic, hierarchical and multipath routing protocols, as shown in Section 6. Section 7 summarizes the most significant schemes for routing protocols defined in Spain, focusing on our contributions. Finally, Section 8 draws the main conclusions of this work.

## 2. Algorithm Paradigms for Wireless Sensor Networks

Sensor applications demand the communication of nodes to execute certain procedures or algorithms. In fact, three kinds of algorithms can be executed on wireless sensor networks [4]:

- *Centralized Algorithms*: They are executed in a node that posses the knowledge of the whole network. These algorithms are quite rare because of the cost of transmitting the data to make the node know the status of the complete network.
- *Distributed Algorithms*: The communication is supported by message-passing.
- *Local based Algorithms*: The nodes use restricted data acquired from a close area. With this local information, the algorithm is executed in one node.

The algorithm paradigm is an important factor to take into account when deciding about the routing protocol to employ in the network. If localized algorithms are used, the routing protocol should reinforce and optimize the communication between neighbors. On the other hand, for centralized algorithms, combining the messages that simultaneously go the central node (even when they are generated by different sources) could be an advantage. The distributed algorithms should efficiently support the communication between any two pairs of nodes. Finally, local based algorithms depend on some solution that provides geographic coordinates, like GPS, making the solution more expensive.

## 3. Design Constraints for Routing in Wireless Sensor Networks

Due to the reduced computing, radio and battery resources of sensors, routing protocols in wireless sensor networks are expected to fulfill the following requirements [5]:

- *Autonomy*: The assumption of a dedicated unit that controls the radio and routing resources does not stand in wireless sensor networks as it could be an easy point of attack. Since there will not be any centralized entity to make the routing decision, the routing procedures are transferred to the network nodes.
- *Energy Efficiency*: Routing protocols should prolong network lifetime while maintaining a good grade of connectivity to allow the communication between nodes. It is important to note that the battery replacement in the sensors is infeasible since most of the sensors are randomly placed. Under some circumstances, the sensors are not even reachable. For instance, in wireless underground sensor networks, some devices are buried to make them able to sense the soil [6].
- *Scalability*: Wireless sensor networks are composed of hundred of nodes so routing protocols should work with this amount of nodes.
- *Resilience*: Sensors may unpredictably stop operating due to environmental reasons or to the battery consumption. Routing protocols should cope with this eventuality so when a current-in-use node fails, an alternative route could be discovered.
- *Device Heterogeneity*: Although most of the civil applications of wireless sensor network rely on homogenous nodes, the introduction of different kinds of sensors could report significant benefits. The use of nodes with different processors, transceivers, power units or sensing components may improve the characteristics of the network. Among other, the scalability of the network, the energy drainage or the bandwidth are potential candidates to benefit from the heterogeneity of nodes [7].
- *Mobility Adaptability*: The different applications of wireless sensor networks could demand nodes to cope with their own mobility, the mobility of the sink or the mobility of the event to sense. Routing protocols should render appropriate support for these movements.

## 4. Classification of Routing Protocols in Wireless Sensor Networks

Taking into account their procedures, routing protocols can be roughly classified according to the following criteria.

### 4.1. Hierarchy Role of Nodes in the Network

In the flat schemes, all sensor nodes participate with the same role in the routing procedures. On the other hand, the hierarchical routing protocols classify sensor nodes according to their functionalities [8]. The network is then divided into groups or clusters. A leader or a cluster head is selected in the group to coordinate the activities within the cluster and to communicate with nodes outside the own cluster. The differentiation of nodes can be static or dynamic.

### 4.2. Data Delivery Model

Depending on the application, data gathering and interaction in wireless sensor networks could be accomplished on several ways. The data delivery model indicates the flow of information between the sensor nodes and the sink [7]. The data delivery models are divided into the following classes: continuous, event-driven, query-driven or hybrid. In the continuous model, the nodes periodically transmit the information that their sensors are detecting at a pre-specified rate. In contrast, the query-driven approaches force nodes to wait to be demanded in order to inform about their sensed data. In the event-driven model, sensors emit their collected data when an event of interests occurs. Finally, the hybrid schemes combine the previous strategies so sensors periodically inform about the collected data but also response to queries. Additionally, they are also programmed to inform about events of interest.

## 5. Optimization Techniques for Routing in Wireless Sensor Networks

The particular characteristics of wireless sensor networks and their constraints have prompted the need for specific requirements to routing protocols. When compared to mobile *ad hoc* networks routing protocols, the algorithms in wireless sensor networks usually realize the following specifications:

### 5.1. Attribute-based

In these algorithms, the sink sends queries to certain regions and waits for the response from the sensors located in this area. Following an attribute-value scheme, the queries inform about the required data. The selection of the attributes depends on the application. An important characteristic of these schemes is that the content of the data messages is analyzed in each hop to make decisions about routing.

### 5.2. Energy Efficiency

Multiple routes can communicate a node and the sink. The aim of energy-aware algorithms is to select those routes that are expected to maximize the network lifetime. To do so, the routes composed of nodes with higher energy resources are preferred.

### 5.3. Data Aggregation

Data collected in sensors are derived from common phenomena so nodes in a close area usually share similar information. A way to reduce energy consumption is data aggregation. Aggregation consists of suppressing redundancy in different data messages. When the suppression is achieved by some signal processing techniques, this operation is called data fusion.

### 5.4. Addressing Scheme

Wireless sensor networks are formed by a significant number of nodes so the manual assignation of unique identifiers is infeasible. The use of the MAC address or the GPS coordinates is not recommended as it introduces a significant payload [3]. However, network-wide unique addresses are not needed to identify the destination node of a specific packet in wireless sensor networks. In fact, attribute-based addressing fits better with the specificities of wireless sensor networks. In this case, an attribute such as node location and sensor type is used to identify the final destination. Concerning these identifiers, two different approaches have been proposed [3]. Firstly, the ID reuse scheme allows identifiers to be repeated in the network but keeping their uniqueness in close areas. In this way, a node knows that its identifier is unique in a *k*-hop neighborhood, being *k* a parameter to configure. On the other hand, the field-wide unique ID schemes guarantee that the identifiers are unique in the whole application. With this assumption, other protocols such as routing, MAC or network configurations can be simultaneously used.

### 5.5. Location-based

When this technique is used, a node decides the transmission route according to the localization of the final destination and the positions of some other nodes in the network.

### 5.6. Multipath Communication

With this technique, nodes use multiple paths from an origin to a destination in the network. As multipath communications are intended to increase the reliability and the performance of the network, these paths should not share any link. Multipath communications can be accomplished in two ways. Firstly, one path is established as the active communication routing while the other paths are stored for future need, i.e. when the current active path is broken. On the other hand, it is also possible to distribute the traffic among the multiple paths.

### 5.7. Quality of Service

**Figure 1.**
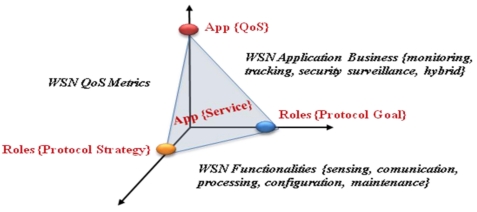
Relation of QoS and Routing Protocol Goal and Strategy.

The network application business and its functionalities prompt the need for ensuring a QoS (Quality of Service) in the data exchange. In particular, effective sample rate, delay bounded and temporary precision are often required. Satisfying them is not possible for all the routing protocols as the demands may be opposite to the protocol principles. For instance, a routing protocol could be designed to extend the network lifetime while an application may demand an effective sample rate which forces periodic transmissions and, in turn, periodic energy consumptions. Figure 1 shows the relation of QoS and its dependence to the routing protocol goal and to the routing protocol strategy.

## 6. Application of the Optimization Techniques: Routing Protocols

By means of representative routing protocols, we present how the attribute-based, the geographic and the multipath techniques are usually applied into wireless sensor networks. Although the hierarchy is commonly considered a parameter for the classification of protocols, we will study it as an important technique used in routing protocols and therefore, we will also analyze some representative hierarchical routing protocols.

### 6.1. Attribute-based or Data-centric Routing Protocols

In this category, the following protocols stand out:

#### 6.1.1. SPIN (Sensor Protocols for Information via Negotiation)

In [9] the authors present a family of adaptive protocols, called SPIN (Sensor Protocols for Information via Negotiation), that efficiently disseminate information among sensors in an energy-constrained wireless sensor network. Nodes running a SPIN communication protocol name their data using high-level data descriptors, called meta-data. They use meta-data negotiations to eliminate the transmission of redundant data throughout the network. In addition, SPIN nodes can base their communication decisions both upon application-specific knowledge of the data and upon knowledge of the resources that are available to them. This allows the sensors to efficiently distribute data given a limited energy supply. Four specific SPIN protocols were simulated and analyzed: SPIN-PP and SPIN-EC, which are optimized for a point-to-point network, and SPIN-BC and SPIN-RL, which are optimized for a broadcast network.

In point-to-point networks, the sender announces that it has new data with an advertisement message to each neighbor. When the neighbor receives the message, the node checks the metadata to know if it already stores the data item. If the neighbor is interested in the information, it responds with a request message. Upon receiving it, the sender transmits the information in a data message. The neighbor that receives the data, inform about its availability to its own neighbors with an advertisement message. The three-handshake protocol is then repeated. The described process is known as SPIN-PP. The algorithm SPIN-EC introduces a technique in the nodes so when their current energy resources do not exceed a predetermined threshold that allows them to complete the three hand-shake protocol, they do not participate in the process. The SPIN-BC and SPIN-RL variants extend the algorithm to support broadcast transmissions. In this way, one advertisement message can reach all the neighbors. In this case, the neighbors do not respond immediately with a request message but they must wait a random time. To optimize the process, a node different from the advertising one cancels its own request message when it detects another similar message. Taking into account the broadcast transmission, the advertising node also responds with just one data message even when it has received multiple request messages. Additionally, SPIN-RL incorporates some reliability functionalities. Specifically, nodes keep track of the advertisement messages that they receive and their corresponding originators. If they send a request message, but the announcing node does not respond in a given interval, the node asks again for the data with a request message.

Comparing the SPIN protocols to other possible approaches, the SPIN protocols can deliver 60% more data for a given amount of energy than conventional approaches in a point-to-point network and 80% more data for a given amount of energy in a broadcast network. In addition, in terms of dissemination rate and energy usage, the SPIN protocols perform close to the theoretical optimum in both point-to-point and broadcast networks. One of the major advantages of these protocols is that nodes are only required to know its 1-hop neighborhood.

#### 6.1.2. Directed Diffusion

As a data-centric protocol, applications in sensors label the data using attribute-value pairs. A node that demands the data generates a request where an interest is specified according to the attribute-value based scheme defined by the application. The sink usually injects an interest in the network for each application task [10]. The nodes update an internal interest cache with the interest messages received. The nodes also keep a data cache where the recent data messages are stored. This structure helps on determining the data rate. On receiving this message, the nodes establish a reply link to the originator of the interest. This link is called gradient and it is characterized by the data rate, duration and expiration time. Additionally, the node activates its sensors to collect the intended data. The reception of an interest message makes the node establish multiple gradients (or first hop in a route) to the sink. In order to identify the optimum gradient, positive and negative reinforcements are used. There algorithm works with two types of gradients: exploratory and data gradients. Exploratory gradients are intended for route set-up and repair whereas data gradients are used for sending real data.

#### 6.1.3. Rumor

In this algorithm, the queries generated by the sink are propagated among the nodes that have observed an event related to the queries [11]. To do so, a node that observes an event inject a long-lived packet called agent. The agents are propagated in the network so distant nodes have knowledge about which nodes have perceived certain events. To optimize the behavior of agents, when an agent reaches a node which has detected another event, the agent is still forwarded but aggregating the new discovered event. Additionally, the agents maintain a list of the recent visited nodes so loops are partially avoided.

On reception of agents, nodes can acquire updated information about the events in the network. This knowledge is reflected in the node's event caches. By using the event cache, a node can conveniently send a query message. However, some nodes may not be aware of the event's originator. Under these circumstances, the query is sequentially propagated to one of the neighbors selected randomly. Once the query arrives at a node with an entry related to the demanded event in its event cache, the query is then forwarded through the learnt path. Following this procedure, the cost of flooding the network with the query is clearly suppressed.

#### 6.1.4. COUGAR

Under this approach, the network is foreseen as a distributed database where some nodes containing the information are temporary unreachable [12]. Since node stores historic values, the network behaves as a data warehouse. Additionally, it is worth noting that poor propagation conditions may lead to the storage of erroneous information in the nodes. Taking into account this circumstance, COUGAR provides a SQL-like interface extended to incorporate some clauses to model the probability distribution. The sink is responsible for generating a query plan which provides the hints to select a special node called the leader. The network leaders perform aggregation and transmit the results to the sink.

#### 6.1.5. ACQUIRE (Active Query Forwarding in Sensor Networks)

This algorithm [13] also considers the wireless sensor network as a distributed database. In this scheme, a node injects an active query packet into the network. Neighboring nodes that detects that the packet contains obsolete information, emits an update message to the node. Then, the node randomly selects a neighbor to propagate the query which needs to resolve it. As the active query progress through network, it is progressively resolved into smaller and smaller components until it is completely solved. Then, the query is returned back to the querying node as a completed response.

### 6.2. Geographical Routing Protocols

These algorithms take advantage of the location information to make routing techniques more efficient. Specifically, neighbors exchange information about their location so when a node needs to forward a packet, it sends it to the neighbor which is assumed to be closest to the final destination. To operate, the source inserts the destination's coordinates in the packets. The location information used in geographical algorithms can be derived from specific devices such as GPS or it can be modeled by virtual coordinates [14].

Concerning geographical protocols, geocasting is the process by which a packet is delivered to the nodes placed in an area. This primitive is especially suitable in wireless sensor networks since the sink usually demands information from the nodes that are in a zone. The zone can be statically determined by the source node or it can be constructed dynamically by the relaying nodes in order to avoid some nodes that may cause a detour.

On the other hand, in geographic-based rendezvous mechanisms, geographical locations are used as a rendezvous place for providers and seekers of information. Geographic-based rendezvous mechanisms can be used as an efficient means for service location and resource discovery, in addition to data dissemination and access in wireless sensor networks [15]. The most popular forwarding techniques in geographical routing protocols are:

#### 6.2.1. Greedy Algorithms

Under this approach, a node decides about the transmission path based on the position of its neighbors. To proceed, the source compares the localization of the destination with the coordinates of its neighbors. Then, it propagates the message to the neighbor which is closest to the final destination. The process is repeated until de packet reaches the intended destination. Several metrics related to the concept of closeness have been proposed for this context. Among them, the most popular metrics are the Euclidean distance and the projected line joining the relaying node and the destination.

With this strategy, flooding processes are restricted to one-hop and the network is able to adapt proficiently to the topological changes. This simple forwarding rule is modified according to the reliability of links in [16]. In this proposal, the unreliable neighbors are not taken into account for the retransmissions. On the other hand, the geographic information is also used in SPEED (Stateless Protocol for End-to-End Delay) to estimate the delay of the transmitted packets [17].

Similar to this algorithm, the greedy algorithm with the ‘most-forward-within-R’ forwarding technique opts to select the most distant neighbor of the packet holder which is closer to the final destination as the next hop [7]. In contrast, the ‘nearest-forward-process’ chooses the nearest neighbor that is closer to the intended destination as the next relaying node.

The main limitation of the greedy algorithms is that the transmission may fail when the current holder of the message has no neighbors closer to the destination than itself. This could occur even when there is a feasible path between the two extremes, for instance, when an obstacle is present. Aiming at overcoming this drawback, the ‘right hand’ rule is suggested [18].

#### 6.2.2. GAF (Geographic Adaptive Fidelity)

This protocol aims at optimizing the performance of wireless sensor networks by identifying equivalent nodes with respect to forwarding packets [19]. Two nodes are considered to be equivalent when they maintain the same set of neighbor nodes and so they can belong to the same communication routes. Source and destination in the application are excluded from this characterization. To identify equivalent nodes, their positions are necessary. Additionally, a virtual grid is constructed. This grid is formed by cells whose size allows to state that all the nodes in one cell can directly communicate with the nodes belonging to adjacent cells and *vice versa*. In this way, the nodes in a cell are equivalent.

Nodes identify equivalent nodes by the periodic exchange of discovery messages with the nodes in their cells. With the information contained in these messages, the nodes negotiate which one is going to support the communications. The other nodes will stay powered off. With this procedure, the routing fidelity is kept, that is, there is uninterrupted connectivity between communicating nodes. However, the elected node periodically rotates for fair energy consumption. To do so, the nodes wake up periodically.

### 6.3. Hierarchical Routing Protocols

The main objective of hierarchical routing is to reduce energy consumption by classifying nodes into clusters. In each cluster, a node is selected as the leader or the cluster head. The different schemes for hierarchical routings mainly differ in how the cluster head is selected and how the nodes behave in the inter and intra-cluster domain.

#### 6.3.1. LEACH (Low Energy Adaptive Clustering Hierarchy)

In LEACH the role of the cluster head is periodically transferred among the nodes in the network in order to distribute the energy consumption. The performance of LEACH is based on rounds. Then, a cluster head is elected in each round. For this election, the number of nodes that have not been cluster heads and the percentage of cluster heads are used. Once the cluster head is defined in the setup phase, it establishes a TDMA schedule for the transmissions in its cluster [20]. This scheduling allows nodes to switch off their interfaces when they are not going to be employed. The cluster head is the router to the sink and it is also responsible for the data aggregation. As the cluster head controls the sensors located in a close area, the data aggregation performed by this leader permits to remove redundancy.

A centralized version of this protocol is LEACH-C [21]. This scheme is also based on time rounds which are divided into the set-up phase and the steady-phase. In the set-up phase, sensors inform the base station about their positions and about their energy level. With this information, the base station decides the structure of clusters and their corresponding cluster heads. Since the base station posses a complete knowledge of the status of the network, the cluster structure resulting from LEACH-C is considered an optimization of the results of LEACH.

#### 6.3.2. PEGASIS (Power-Efficient Gathering in Sensor Information Systems)

It is considered an optimization of the LEACH algorithm. Rather than classifying nodes in clusters, the algorithm forms chains of the sensor nodes. Based on this structure, each node transmits to and receives from only one closest node of its neighbors. With this purpose, the nodes adjust the power of their transmissions [22]. The node performs data aggregation and forwards it the node in the chain that communicates with the sink. In each round, one node in the chain is elected to communicate with the sink. The chain is constructed with a greedy algorithm.

#### 6.3.3. TEEN (Threshold Sensitive Energy Efficient Sensor Network Protocol)

TEEN [23] is other hierarchical protocol for reactive networks that responds immediately to changes in the relevant parameters. In this protocol a clusters head (CH) sends a hard threshold value and a soft one. The nodes sense their environment continuously. The first time a parameter from the attribute set reaches its hard threshold value, the node switches on its transmitter and sends its data. The nodes then transmits data in the current cluster period if the following conditions are true: the current value of the sensed attribute is greater than the hard threshold, and the current value of the sensed attribute differs from sensed value by an amount equal to or greater than the soft threshold. Both strategy looks to reduce energy spend transmitting messages.

The main drawback of this scheme is that, if the thresholds are not reached, the nodes will never communicate; the user will not get any data from the network at all and will not come to know even if all the nodes die. Thus, this scheme is not well suited for applications where the user needs to get data on a regular basis.

#### 6.3.4. DirQ (Directed Query Dissemination)

DirQ [24] aims at optimizing the propagation of queries in a wireless sensor network. The main objective is that the queries are just propagated by the minimum number of nodes that ensure that the queries arrive at the nodes that are able to service the query. To do so, certain information is exchanged in the network. The periodicity of the update messages depend on the rate of variation of the physical parameters that the network is sensing. Then, each node autonomously maintains its own threshold (*δ*). If a sensor node has a value *V* of a desired parameter and the next measurement period gets the same or a similar value in the interval between (*δ* − *V*, *V* + *δ*) then it decides not to send anything to sink. However, if the sink does not receive any message from a specific node then it assumes that this node has a measured value that has not changed much from what has been reported recently. To allow a precise delivery of applications, all network nodes must be capable of storing information which can be considered a disadvantage depending on the amount of information stored in the topology and the number of nodes. DirQ is a protocol suitable for situations where the number of requests is high and times of transmission of requests are known.

### 6.4. Multipath Routing Protocols

In these protocols, a source knows multiple routes to a destination. The routes can be simultaneously used or one of them can be active while the others are maintained for future needs.

#### 6.4.1. SAR (Sequential Assignment Routing)

SAR [25] is one of the first protocols for wireless sensor networks that provide the notion of QoS routing criteria. It is based on the association of a priority level to each packet. Additionally, the links and the routes are related to a metric that characterizes their potential provision of quality of service. This metric is based on the delay and the energy cost. Then, the algorithm creates trees rooted at the one-hop neighbors of the sink. To do so, several parameters such as the packet priority, the energy resources and the QoS metrics are taken into account. The protocol must periodically recalculate the routes to be prepared in case of failure of one of the active nodes.

#### 6.4.2. Maximum Lifetime Routing in Wireless Sensor Networks

This algorithm combines the energy consumption optimization with the use of multiple routes [26]. In this algorithm an active route (also called the primary route) is monitored to control its residual energy. Meanwhile other routes can be discovered. If the residual energy of the active route does not exceed the energy of an alternative route, the corresponding secondary route is then used.

#### 6.4.3. Energy Aware Routing in Wireless Sensor Networks

Once multiple paths are discovered, this algorithm associates a probability of use to each route [27]. This probability is related to the residual energy of the nodes that form the route but it is also considers the cost of transmitting through that route.

#### 6.4.4. M-MPR (Mesh Multipath Routing)

This protocol presents two operation modes [28]. Firstly, in the disjoint MPR (D-MPR) with Selective Forwarding each packet is individually analyzed by the source and it is routed through different routes. Secondly, the D-MPR with data replication is based on the simultaneous emission of multiple copies of the same packet through different routes. Specifically, all the known routes that communicate the source and the destination propagate the packet. For the route discovery, information about the position of the nodes and about their residual energy is exchanged.

### 6.5. Comparison

Hierarchical and geographic routing protocols are considered scalable solutions. Keeping a hierarchical structure demands the coordination of nodes by means of transmitted messages. In dense networks, the use of the cluster-based structure makes up for this cost. However, this benefit does not hold in small networks. A similar behavior is observed for geographic approaches. When the network is composed of a significant number of nodes in an extended area, the exchange of messages to establish the location of neighbors becomes negligible compared to the reduction of transmissions that the geographic algorithm achieves. In these two approaches, the topology of the network must be stable. On the contrary, the cluster structure and the geographic information must be frequently updated which leads to additional costs. For instance, in stable networks, PEGASIS is usually more efficient than LEACH. However, the construction of the chains in PEGASIS could lead to significant resource consumption in highly dynamic topologies.

Attribute-based techniques become relevant when the data sensed by the nodes are not usually of interest to the rest of the nodes. Under these circumstances, the algorithms could greatly reduce the network overhead. The decision about which algorithm should be selected mainly depends on the data delivery model that the application forces. When the communication should be triggered by events, SPIN is the most suitable attribute-based algorithm. However, Directed Diffusion, Rumor, COUGAR and ACQUIRE are query-driven protocol. They roughly differ in how the query is propagated and resolved in the network.

Concerning the multipath routing protocols, their main disadvantage lies on the cost of maintaining the paths. This cost comprises memory resources as well as network overhead. Therefore, they are not appropriate for networks critically constrained by their reduced batteries. However, they become necessary when reliability is a strong requirement in the application business.

## 7. Routing Protocols proposed by Spanish Universities

Among these studies, the following works stand out:

### 7.1. Beacon-less Geographic Routing Protocols

The geographic routing protocols were initially conceived to operate with the periodic exchange of messages that inform about the position of nodes in the network. These messages or beacons incurs in an additional overhead, which represents the main disadvantage of this kind of protocols. In [29] the suitability of suppressing the beacon messages in the geographic routing protocols [29] is analyzed. The beacon-less algorithms are then supported by the reactive exchange of location information just when the nodes need to route data. The paper analyzes five beacon-less routing protocols: IGF (Implicit Geographic Forwarding), GeRaF (Geographic Random Forwarding), CBF (Contention Based Forwarding), BLR and BOSS, which was proposed by the group.

### 7.2. QoS Routing Protocols based on Artificial Intelligence

In [30] a routing protocol that guarantees some QoS requirements by means of an artificial intelligence technique is presented. Neural networks are then introduced into the sensor nodes and a self-organized map is used. The simulation results show its ability to reduce the end-to-end delay and the network overhead compared to the Directed Diffusion protocol.

### 7.3. Energy Aware Routing Protocols for Underwater Sensor Networks

Underwater wireless sensor networks consist of a certain number of sensors and vehicles that interact to collect data and perform collaborative tasks. Designing energy-efficient routing protocols for this type of networks is essential and challenging because sensor nodes are powered by batteries, which are difficult to replace or recharge, and because underwater communications are severely affected by network dynamics, large propagation delays and high error probability of acoustic channels. In [31] the authors analyze the total energy consumption in underwater acoustic sensor networks considering two different scenarios: shallow water and deep water. Specifically, the direct transmission, the relaying scheme and the clustering structure are compared. They conclude that the worst performance is obtained by the direct communications. For shallow water, the clustering scheme is the best option in terms of network overhead, the end-to-end delay and overhead. Additionally, its scalability in terms of number of nodes and distance is demonstrated.

### 7.4. SHRP (Simple Hierarchical Routing Protocol)

In a research project supported by Petróleos de Venezuela, S.A (PDVSA), the state-owned corporation of the Bolivarian Republic of Venezuela responsible for the efficient, profitable, and dependable exploration, production, refining, transport and commerce of hydrocarbons, our group was interested in considering a routing protocol that could deal with three different aspects: battery availability, number of hops and link quality to guarantee the arrival of messages in the sink node in a energy saving way. We could not find any WSN routing protocol that used these three parameters together and at the same time was concerned about energy saving and reliability features. For this reason a new routing protocol called SHRP was proposed [32].

The SHRP protocol is concerned with topology maintenance that is directed related to the reliability of data delivery. To arrange this it makes use of metrics like local battery availability and link quality between neighbor nodes in choosing the best route into the sink node.

It is also concerned with energy saving as not all periodic data are sent to the sink, as there is a concern that transmission is the task that more wastes energy in wireless sensor networks [33]. The SHRP protocol just sends data that have not changed from the last sensing data. The coordinators nodes can aggregate various data messages and send just one message. In this manner SHRP protocol has also an energy saving behavior.

SHRP provides a load balance scheme during the creation of best routes groups, so not always will the same best route be chosen.

To be flexible and to contribute with new metrics for others routing protocols, the SHRP architecture uses SP [33], a specific unifying protocol of TinyOS operating system. SP allows network protocols to choose neighbors wisely, taking into account information available at the link layer, providing a great modularity. The SHRP protocol periodically monitors the battery lifetime and link quality, cutting off from the routing table nodes that can not contribute in maintaining a well connected topology.

The proposed protocol takes care of link quality, cutting off neighbor nodes from the routing table nodes that have the average link quality indicator below a minimum threshold. This threshold is related to the IEEE LQI indicator [34]. The SHRP protocol also cuts off from the routing table neighbor nodes that have the RSSI (Received Signal Strength Indicator) values below a minimum threshold [35]. Also, the SHRP protocol cuts off nodes that do not have battery availability, at least to execute what we call “*Minimum Task Cycle*” (MTC) [36] (see Equation 1).

(1) MTC=CCA+Sensing Task+Transmission Task+Reception Task+Idle Period Task

All the tasks of cutting off neighbor nodes, shown before, represent that if a node does not have sufficient battery power or has a bad link quality, caused by interference, multipath or path loss it will not participate as a route node in the choosing of the best route.

To provide the topology maintenance SHRP protocol defines new metrics that SP does not provide. To be aware about the state of the energy of the nodes, SHRP defines a metric called “minbattrem” that keeps information about the energy available in each node along each route until the sink node.

SHRP protocol tries to choose the route that gives more reliability, this mean, the route that give the mayor energy available along all the possible routes until the sink node.

Another metric defined by SHRP protocol is called “nhops”. With this metric SHRP can choose a route that takes into account the number of hops to the sink node. As the transmission is the task that uses more energy, SHRP tries to choose the route that pass through the minor number of hops, saving more energy.

Newer radios that are based on IEEE 802.15.4 standard such as CC2420 implement a parameter called link quality indicator (LQI) which is believed to be a better indicator than RSSI [20]. SHRP protocol uses the metric called “AvgLQI” that represents this parameter.

Protocol designers looking for inexpensive and agile link estimators may choose RSSI over LQI [34], but RSSI at the edge of the threshold of -87 dBm does not have a good correlation with PRR, so SHRP uses both of the metrics, LQI and RSS, in choosing the best route. LQI and RSSI metrics are obtained from the link layer of the wireless network. In our experiments we used the CC2420 radio chip.

**Figure 2.**
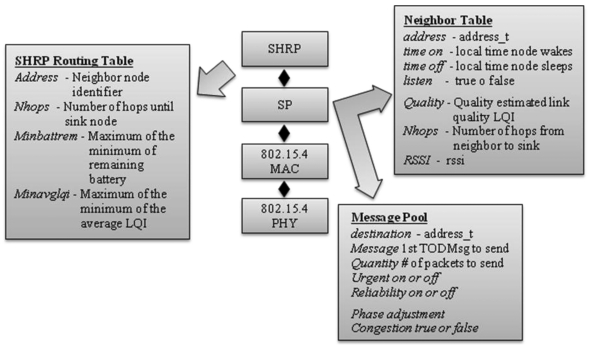
Interaction between SHRP protocol and SP.

Figure 2 shows the interaction of SHRP protocol with SP to get information about neighbor nodes and to decide about the best route into the sink node. The metrics shown in bold font are the new ones defined by SHRP protocol. In this manner, other protocol that makes use of any of these metrics will have them already available in SP module.

The SHRP protocol defines three kinds of sensing data messages: (i) periodic; (ii) alert; (iii) alarm. The sink node sends the query message, that is explained later, with the information that the monitoring system is interested to collect periodically. Based on this message, the sensing nodes know what and when they have to sense.

**Figure 3.**
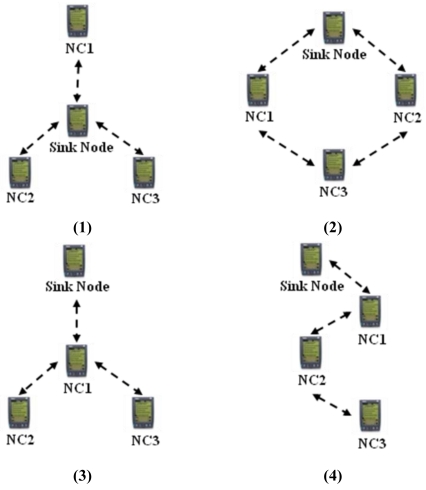
Topologies of the Experiments.

The alert message is sent when the sensed data value is above an average value, specified by the monitoring system. The alarm message is sent when the value of the data sensed is below a minimum or above a maximum threshold, also specified by the system. This message is sent with the Urgent and Reliability bits ‘on’, so they have priority over any other message and the sender waits for an ACK message, to confirm that the alarm message have arrived. To do some experimental tests was defined the ‘convergence time’ as a period that takes long for all NC nodes update its routing tables, so it includes all transmission and processing times. In a Wireless Sensor Network, the clocks that come with each mote works independently, so in order to calculate the convergence time of SHRP protocol, was implemented a synchronization mechanism based on the TPSN scheme. The experimental tests were done in four different topologies (see Figure 3) to observe the worst case (maximum) of convergence time. Was repeated five times each experiment. Tests were done using TMote motes from the Moteiv Company with the TinyOS operating system and the CC2420 Radio Chip.

**Figure 4.**
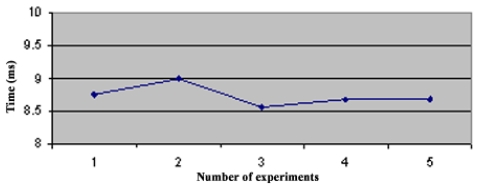
Convergence Time of Topology 1.

**Figure 5.**
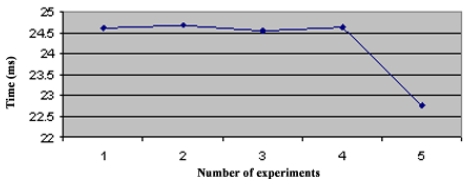
Convergence Time of Topology 2.

**Figure 6.**
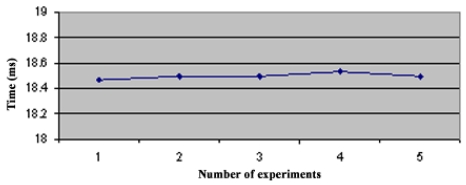
Convergence Time of Topology 3.

**Figure 7.**
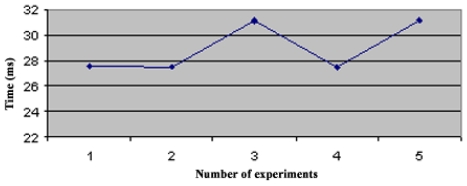
Convergence Time of Topology 4.

In the star topology (see Figure 3.1) all NC nodes have a direct link with the sink node. The results of the convergence time of this topology can be seen on Figure 4. In the topology 3.2 the NC1 and NC2 nodes have a direct link with the sink node, but node NC3 just has a direct link with NC1 and NC2 nodes. With this topology NC3 has a redundant path until sink node and has two ‘father’ nodes (see Figure 5.). As we can notice in Figure 6, topology 3.3, NC2 and NC3 are two hops far from the sink node, having just one ‘father’ node (NC1). In the topology 3.4 (see Figure 7.) there are three hops until sink node with the following direct links: sink-NC1, NC1-NC2 and NC2-NC3, showing a tandem topology.

**Figure 8.**
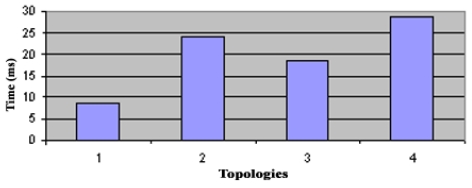
Comparison of Convergence Times.

The consolidation of the data is shown in Figure 8 with the media of the results from each topology. As can be seen, topologies 2, 3 and 4 maintain the same convergence time behavior, increasing in a linear way regarding to the number of hops. This result was obtained due to each NC node has only one neighbor to learn route table. In the second topology, the convergence time had a different behavior when comparing it with the topology 3. Both of them are two hops from the sink node; however the processing time is greater in topology 2 because NC3 node in topology 3 has more neighbors, so NC3 node has to process more NMI messages and calculate more in order to have the best route until sink node.

With these experiments the conclusion is that SHRP protocol has a media convergence time of 8 ms for each direct link that has to exchange NMI messages, and this time increases linearly with the increase of neighbor nodes.

The second experiments that we have done are the comparison of SHRP protocol and HTS (*Hop to Sink Protocol*) protocol. The HTS protocol is a simple routing protocol that we have proposed and implemented. The only metrics that it uses are the number of hops and the sink node has to receive all routing table from all nodes to decide the best route and send the decision to each node. HTS protocol looks like a link state protocol but just sink node knows all routes from all NC nodes. In this study there are the same topologies shown in Figure 4 with the same parameters previously described.

**Figure 9.**
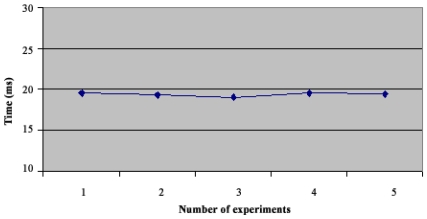
Convergence Time of Topology 1.

**Figure 10.**
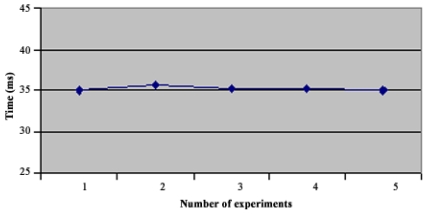
Convergence Time of Topology 2.

**Figure 11.**
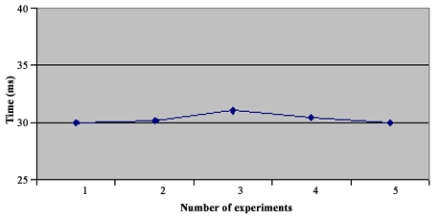
Convergence Time of Topology 3.

As we can see in Figure 9, the convergence time for the first topology reached 20 ms. With SHRP we had a maximum convergence time of 9 ms. Now in topology 2 (Figure 10), the maximum Convergence Time was 35 ms, meanwhile with SHRP protocol it was 24.7 ms. In Figure 11 is shown the Convergence Time of HTS in topology 3. As can be seen the maximum Convergence Time was 31 ms and with SHRP protocol it was 18.5 ms.

These experiments shows that SHRP protocol has a better convergence time with respect to a simple protocol such as HTS, that just chooses the neighbors based on the number of hops to the sink node. Link state protocols like HTS, where each node has to know all the routes to the sink node, can have a large convergence time, being a problem when there is a topology that changes constantly as in wireless sensor network, as we have low battery time, interference and obstacles problems. So we can conclude that protocols that just need to have neighbor information to decide the best route shows to have a lower convergence time, being more interesting to WSN.

## 8. Conclusions

The proliferation of smart, light-weight sensors has made wireless sensor network popular. The constrained capabilities of the devices should be taken into account for the development of applications for these networks. Concerning the routing protocols, the reduced energy resources, the scalability and the resilience arise as the main limitations in wireless sensor networks. This paper presents a survey on how routing protocols are adapted to these characteristics. Additionally, the paper includes the efforts carried out by Spanish universities on developing optimization techniques in the area of routing protocols for wireless sensor networks, focusing on our contribution: a proposal of routing protocol for wireless sensor networks, called SHRP (Simple Hierarchical Routing Protocol). Aiming at prolonging network lifetime, this protocol decides the best route according to three different metrics: battery availability, number of hops and link quality. The algorithm also includes a load-balance technique so that the traffic is distributed among the possible routes. To be flexible and to contribute with new metrics for others routing protocols, the SHRP architecture uses SP that allows network protocols to choose neighbors wisely, taking into account information available at the link layer, providing a great modularity.

## Figures and Tables

**Table 1.** Summary of the characteristics of the routing protocols that will be studied in this section. As we can observe, the combination of the optimization techniques is usual.

**Protocol**	**Applied Technique**					
	**Attribute-based**	**Energy-Efficiency**	**Location-based**	**Multipath**	**QoS**	**Hierarchy**
SPIN	Yes					
Directed Diffusion	Yes					
Rumor	Yes					
COUGAR	Yes					
ACQUIRE	Yes					
GAF		Yes	Yes			
LEACH		Yes				Yes
PEGASIS		Yes			Yes	Yes
TEEN		Yes				Yes
DirQ						Yes
SHRP		Yes		Yes	Yes	Yes
SAR				Yes	Yes	
Maximum Lifetime		Yes		Yes		
Energy Aware		Yes		Yes		
M-MPR		Yes	Yes	Yes		
